# In-Line Inspection Tool with Eddy Current Instrumentation for Fatigue Crack Detection

**DOI:** 10.3390/s18072161

**Published:** 2018-07-05

**Authors:** Cesar Camerini, João Marcos Alcoforado Rebello, Lucas Braga, Rafael Santos, Tomasz Chady, Grzegorz Psuj, Gabriela Pereira

**Affiliations:** 1 Laboratory of Nondestructive Testing, Corrosion and Welding, Federal University of Rio de Janeiro, Rio de Janeiro 21941-596, Brazil; lucascampos@poli.ufrj.br (L.B.); gpereira@metalmat.ufrj.br (G.P.); 2 Metallurgy and Materials Department, Federal University of Rio de Janeiro, Rio de Janeiro 21941-596, Brazil; jmarcos@metalmat.ufrj.br; 3 Petrobras R&D Centre, Ilha do Fundão, Rio de Janeiro 21941-970, Brazil; rwfsantos@petrobras.com.br; 4 West Pomeranian University of Technology, Faculty of Electrical Engineering, Sikorskiego 37 St., 70-313 Szczecin, Poland; tchady@zut.edu.pl (T.C.); gpsuj@zut.edu.pl (G.P.)

**Keywords:** clad material, fatigue crack, eddy current testing, in-line inspection tool

## Abstract

Eddy current transducer with sensing coils placed orthogonally and connected in differential mode was introduced to evaluate fatigue cracks in clad pipeline circumferential welds. A dedicated embedded electronic hardware was developed to drive the transducer and measure the electrical complex impedance of the coils, and was specifically designed for operation under autonomous in-line inspection tool. In the laboratory experiments, an automated inspection was performed with the goal to evaluate transducer’s detectability, and different scanning speed was tested to reproduce in service situation. The results have confirmed that the introduced eddy current transducer is a potential solution for fatigue crack detection in clad circumferential weld root, while the hardware developed presented a reasonable SNR reaching the data rate required to be incorporated in an autonomous in-line inspection tool.

## 1. Introduction

The application of clad material to subsea pipelines is gaining ground in deep water oil exploration. Its bimetallic configuration presents an attractive combination of mechanical strength and corrosion resistance, ensuring the safety and integrity of pipelines that connect the reservoir to oil rig. The clad material for oil exploration consists of a base material, usually carbon steel, inner coated with a thin layer of corrosion resistance alloy (CRA), turning into an attractive economical solution for deep water exploration, since only a small portion of the noble anti-corrosive alloy is required. Clad material has a metallurgical bond between the CRA and the base material attained by carbon diffusion during the hot rolling process [1].

**Figure 1:**
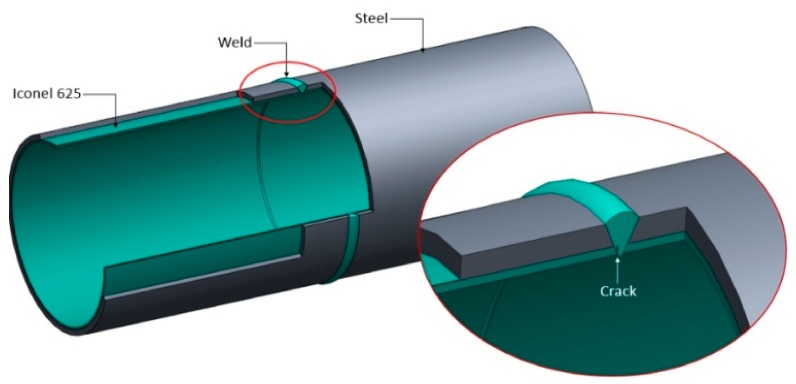
Clad pipe with the inspection region highlighted.

The potential for fatigue cracks to occur in pipeline structures due to cycling loads inherent of offshore oil production (such as, tide variation, waves, ocean current, platform movements, etc.) makes it necessary to have an inspection tool to carry out periodic nondestructive inspection in the inner pipe surface. In case of clad material, it is crucial to detect fatigue crack on its initial stage, because if the crack propagates through the layer of the CRA and reaches the carbon steel, a strong galvanic couple is completed, accelerating, exponentially, the fatigue corrosion process [2]. The most critical point of pipeline structures is the circumferential weld [3], and demands special attention during inspection. Figure 1 presents a section of clad pipeline with a base material of carbon steel API X65 coated with Inconel 625, and highlights the inspection region with the crack positioning.

This scenario, detection of fatigue crack in Inconel (according to its electromagnetic properties), encouraged the development of an eddy current (EC) system for inner inspection of clad pipelines. The main challenges are the circumferential weld geometry and the fact that, generally, in-line inspection tools operate in a speed range between 0.5 and 4.0 m/s [4], which affects, directly, the transducer detection capability and the tool longitudinal resolution.

The techniques instrumented in the commercial in-line inspection tool, such as, MFL (magnetic flux leakage), ultrasound, EMAT (electromagnetic acoustic transducer), are very effective in inspection of generalized corrosion or micro cracks in the base metal of carbon steel pipelines [5,6,7]. However, because of some practical limitation, such techniques are not efficient for detecting micro cracks in welded parts. Reber et al. [8] have shown an ultrasonic configuration for crack detection in carbon steel pipeline girth welds, and presented relevant experimental results demonstrating the technique capability. Nevertheless, the authors highlighted, in their conclusions, that the application of such a technique in in-line inspection tools is still a challenge. Such a challenge gets even more complex in the case of clad material inspection, where the anti-corrosive layer results in an additional interface for the ultrasonic wave propagation, interfering directly in the incident and refracted wave. Moreover, Cheng et al. [9] pointed out that ultrasonic testing is not effective for inspections of Inconel welds, because of its strong inhomogeneity and anisotropy. Once the ultrasound wave is sensitive to grain structures [10], Inconel welds significantly scatter the waves so that clear echoes due to defects cannot always be noticed.

Such challenges motivate the feasibility study of an in-line inspect tool development to detect fatigue cracks in the circumferential welds of clad pipelines based on eddy current concept. Yusa et al. [11,12] and Todorov et al. [13] presented the capability of the EC transducer for fatigue crack detection in welded joints. Among the publications analyzed [11,12,13,14,15,16,17,18], it was verified that the EC transducer with orthogonal configuration of coils exhibits the most significant inspection results. Its differential configuration, and the fact that the coils are located close to each other, minimizes the influence of the weld root profile in the inspection signal. Besides the relevant results completed with the orthogonal transducer, none of the authors have evaluated its behavior and performance when operating at high-speed condition, relevant for field application considering pipeline inspection. In addition, the tests performed in the examined studies used commercial or lab EC equipment, which restricts the application in tools that demand embedded electronic hardware.

The goal of the present work is evaluating the capability of an EC transducer to successfully meet the previously described requirements: detect fatigue cracks in the circumferential weld root of clad pipelines when operating at different inspection speed. An orthogonal coils EC-based transducer was manufactured and tested, and a specific electronic hardware was developed to drive the transducer and measure the testing coils electrical complex impedance.

**Figure 2:**
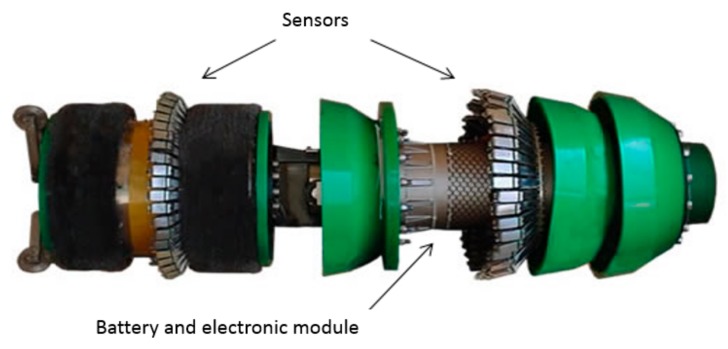
Typical in-line inspection tool for pipeline inspection instrumented with magnetic flux leakage (MFL) technique.

In-line inspection tool, commonly called PIG, is a type of tool widely employed to inner inspect metallic pipelines in different engineering fields. The tool is autonomous and designed to inspect long pipelines, in a range of several kilometers. Normally propelled by the fluid under production, the tool requires a sensor matrix to cover the pipe perimeter, a dedicated electronic hardware to drive, process, and save the sensor data during the inspection process, and a battery module to source the system. Figure 2 presents a typical in-line inspection tool instrument, basing on MFL technique. It is worth mentioning that the present work does not consider the design of an in-line inspection tool for fatigue crack detection in clad pipelines. It focuses primarily in the sensors and hardware development, and in the validation of some specific requirements to demonstrate the feasibility of developing an in-line inspect tool to detect fatigue cracks in the circumferential welds of clad pipelines. In the experimental tests, an array composed of five orthogonal coils, representing the sensor module in a typical in-line inspection tool, scanned a clad material with several scanning speeds. The results achieved demonstrated that the orthogonal EC configuration is a potential solution for fatigue crack detection on circumferential weld root of clad pipelines.

## 2. Materials and Measuring System

**Figure 3:**
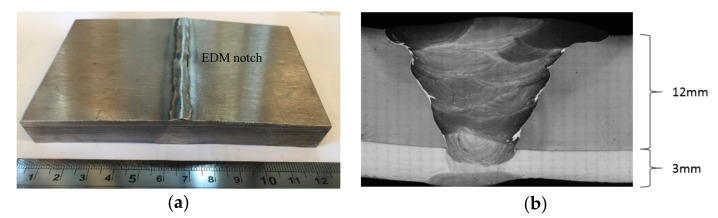
(**a**) Clad sample with the weld bead and electrical discharge machining (EDM) notch; (**b**) metallographic image of the weld cross section.

A clad plate with substrate of carbon steel high strength low alloy, API 5L X65, and clad layer of Inconel 625, with dimensions 120 × 80 × 15 mm, was manufactured with a 45° bevel to receive a weld bead from GTAW (gas tungsten arc welding) weld process. An electrical discharge machining (EDM) notch with dimensions of 10.0 × 1.5 × 0.2 mm was machined in the central part of the Inconel side between the weld root and the Inconel base material. Figure 3a presents a photo of the testing sample with the EDM notch indication, while Figure 3b, a metallographic image of the weld cross section after mechanical grinding, polishing, and etching with chromium nitride solution. One may observe the thickness of the carbon steel layer, 12 mm, and the Inconel 625 clad of 3 mm.

**Figure 4:**
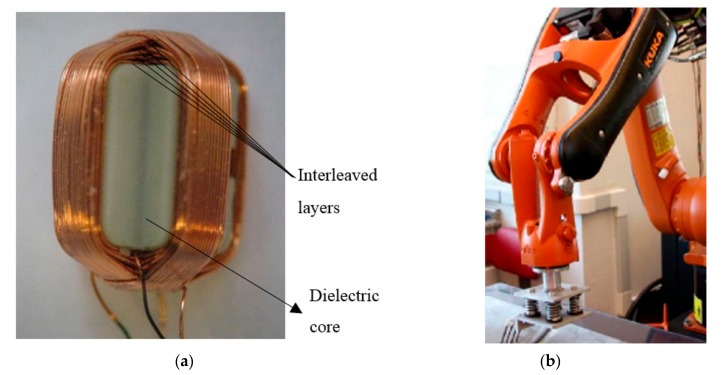
(**a**) Orthogonal coils with the layers interweaved; (**b**) KUKA robotic arm for automated inspection.

The transducer manufactured to inspect the notch consists of the testing coils placed in orthogonal configuration with layers interweaved, wound over a dielectric core, as shown by Figure 4a. The coils are differentially connected, thereby reducing spurious signals caused by lift-off variation during the inspection process [18]. When compared with EC pencil probes, orthogonal coils present low sensitivity to lift-off, allowing reduction of its influence rate from 40 dB/mm to 8 dB/mm. For weld inspection, orthogonal coil configurations present relevant results, because spurious signals arising from some specific materials’ characteristics or from some physical structures that are common to both coils are annihilated, thereby providing no undesirable response. Each manufactured coil present 5 interleaved layers with 15 turns per layer, and an average inductance of 36.1 µH. The transducer testing frequency was 400 kHz. It is worth mentioning that the transducer works in a frequency range of 100–500 kHz, and once the analyzed defects are superficial, higher frequencies result in higher current densities, which increase the inspection sensibility. However, due to hardware limitations, it was not possible to test excitation frequencies above 400 kHz, because of sampling frequency limitation of the digital-to-analog converter (DAC), and the analog-to-digital converter (ADC).

To assist the inspection, a KUKA robotic arm model Hollow Wrist with KRC4 controller was used (Figure 4b). With a payload of five kilograms, the robotic arm carries the sensors and tests different inspection speeds, from 0.05 m/s to 1.0 m/s.

**Figure 5:**
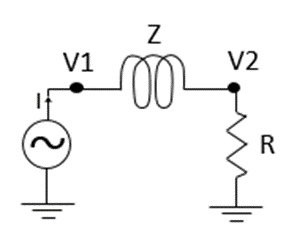
Electrical scheme of a coil.

The electronic hardware was developed to drive the EC sensors and measure the electrical complex impedance of the testing coils. Figure 5 present the basic concept of the EC coils impedance calculation procedure. First, in order to conduct the calculations, both voltage and current of the testing coil were measured. For that purpose, the shunt resistor was utilized and two complex potentials (*V*_1_, and *V*_2_) were sensed. Later, the Ohm’s law in phasor form is applied to obtain the magnitude (Equation (6)) and angle (Equation (7)) of the complex impedance as demonstrated by Equations (1)–(7).

(1) $$V_{1}=\left|V_{1}\right|e^{j\varphi_{1}}=\left|V_{1}\right|\cos\left(\varphi_{1}\right)+j\left|V_{1}\right|\sin\left(\varphi_{1}\right)$$

(2) $$V_{2}=\left|V_{2}\right|e^{j\varphi_{2}}=\left|V_{2}\right|\cos\left(\varphi_{2}\right)+j\left|V_{2}\right|\sin\left(\varphi_{2}\right)$$

(3) $$V_{2}=RI\to \;\frac{V_{2}}{R}=I$$

(4) $$V_{1}-V_{2}=Z\cdot I=\;\frac{Z\cdot V_{2}}{R}$$

(5) $$\frac{R(V_{1}-V_{2})}{V_{2}}=Z$$

(6) $$\left|Z\right|=R\frac{\sqrt{{\left(\left|V_{1}\right|\cos\left(\varphi_{1}\right)-\left|V_{2}\right|\cos\left(\varphi_{2}\right)\right)}^{2}+{\left(\left|V_{1}\right|\sin\left(\varphi_{1}\right)-\left|V_{2}\right|\sin\left(\varphi_{2}\right)\right)}^{2}}}{\sqrt{{\left(\left|V_{2}\right|\cos\left(\varphi_{2}\right)\right)}^{2}+{\left(\left|V_{2}\right|\sin\left(\varphi_{2}\right)\right)}^{2}\;}}$$

(7) $$∠Z=\arctan\left(\frac{\left|V_{1}\right|\sin\left(\varphi_{1}\right)-\left|V_{2}\right|\sin\left(\varphi_{2}\right)}{\left|V_{1}\right|\cos\left(\varphi_{1}\right)-\left|V_{2}\right|\cos\left(\varphi_{2}\right)}\right)-\arctan{\left(\frac{\left|V_{2}\right|\sin\left(\varphi_{2}\right)}{\left|V_{2}\right|\cos\left(\varphi_{2}\right)}\right)}_{.}\;$$

**Figure 6:**
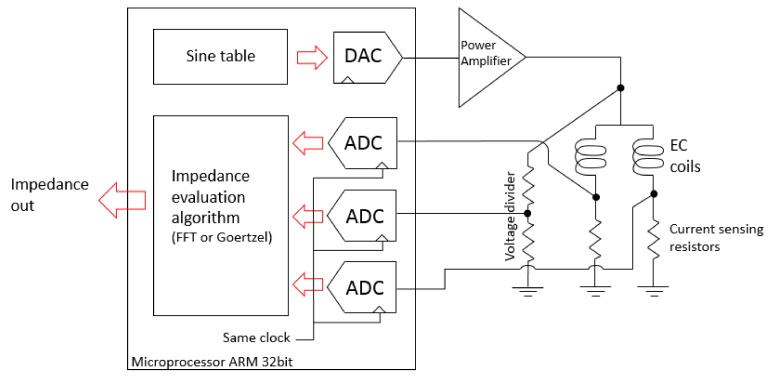
Schematic block of the hardware to drive the eddy current coils and evaluate the impedance variation.

**Figure 7:**
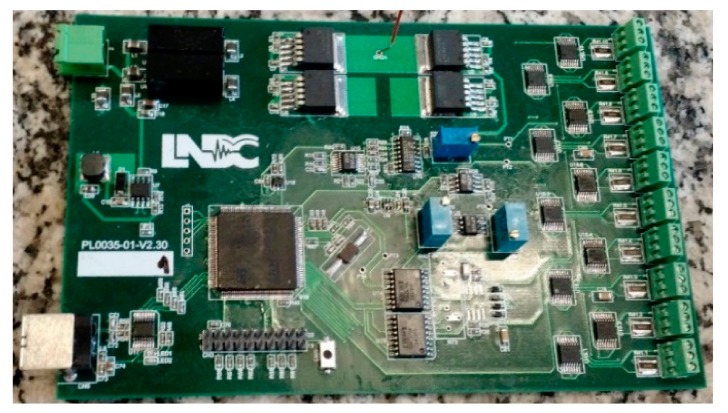
Photo of the electronic hardware developed to drive and measure the EC transducer impedance variation.

Figure 6 presents the block scheme of the measuring system, while Figure 7 shows a photo of the developed electronic printed circuit board (PCB). As shown in the block scheme, the excitation signal is a sine wave with parameters defined in the form of the table stored in the microprocessor ARM 32-bit (STM32F407), which follows to a DAC and a power amplifier based on LT1207, to finally drive the coils. The hardware measures signals that are scaled versions of the voltage over the coils and their currents, and these signals are digitized by an ADC. The STM32F407 internal ADCs and DACs were used as presented in Figure 6. Then, in order to turn the digitized waveforms into phasors, a fast Fourier transform (FFT) or a similar algorithm, allowing processing of the analysis in the frequency domain, is applied.

Initially, the algorithm implemented in the microprocessor to calculate the magnitude and phase of the complex impedance was the fast Fourier transform (FFT). However, in order to improve the hardware processing time and, consequently, the experimental data rate, the FFT was replaced with the Goertzel algorithm. The Goertzel algorithm is an efficient evaluation of individual terms of the discrete Fourier transform (DFT). When the full spectrum analysis needs to be carried out, the Goertzel algorithm is less efficient, because it presents a higher order of complexity than FFT. On the other side, in case of computing a small number of frequency components, it is more numerically efficient (than using the FFT), being very useful for small processors and embedded applications [19]. In the case of conventional EC testing, where the transducer is excited by a single known frequency, the Goertzel algorithm seems to be very suitable to calculate the coil impedance variation.

Equation (8) [19] presents the computed DFT term for the input sequence x[n] in the chosen frequency range $\omega_{0}$ using the Goertzel analysis. The index k indicates the frequency bin of the DFT. If, for instance, a sine wave with 8 points was used, then the 8th bin of the FFT will have the real and imaginary information that can be turned into magnitude and phase. However, if, instead of using FFT to calculate all the bins, it is possible to use the Goertzel algorithm to calculate only *x* [7], where less computational effort is conducted.

(8) $$y\left[N\right]=\overset{N}{\underset{n=0}{\sum }}x\left[n\right]e^{-j\omega_{0}}$$

where $\omega_{0}=2\pi \frac{k}{N}$ and k∈{0,1,2,…,N−1}.

**Figure 8:**
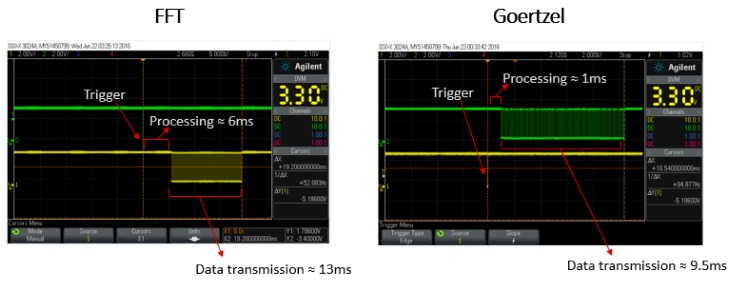
Oscilloscope images showing the time processing achieved by fast Fourier transform (FFT) and Goertzel algorithm.

The use of the Goertzel algorithm offered a significant improvement in the hardware data streaming. In comparison to the FFT, the Goertzel analysis resulted in a calculation speed that was six times faster. On the other hand, while it provides only a single term of the DFT, some relevant information, especially concerning harmonics content, is lost. If the excitation signal saturates the ADC, it can be easily noticed by the distortion caused in the FFT spectrum, and can be evaluated based on the harmonics analysis. In such cases, the total harmonic distortion (THD) coefficient can be used. It defines the ratio between main and other harmonics, and gives evidence about the behavior of the coil input excitation signal. However, according to the properties of the Goertzel algorithm, the calculation of THD is then limited. Nevertheless, it was decided to work with the faster algorithm, in order to increase the hardware data streaming, which is quite relevant for high-speed application. Figure 8 presents two oscilloscope images of the time required for each algorithm implemented in the microprocessor unit to calculate the complex impedance of the coils.

Finally, the faster calculation and less data in the streaming package (THD data is only calculated in the FFT version) allowed the total data rate of the electronic hardware to be increased from ≈50 Hz (1/10.5 ms) for FFT to ≈100 Hz (1/19 ms) for Goertzel.

## 3. Results and Discussion

**Figure 9:**
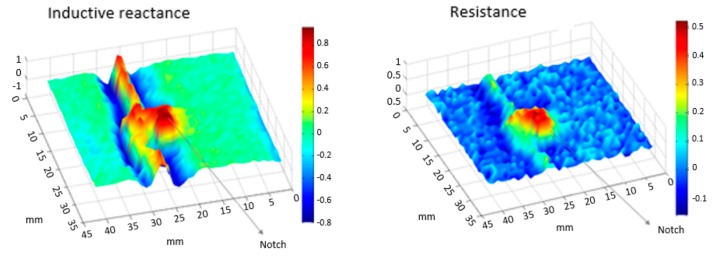
Inspection result of the clad sample with the notch besides the weld bead.

**Figure 10:**
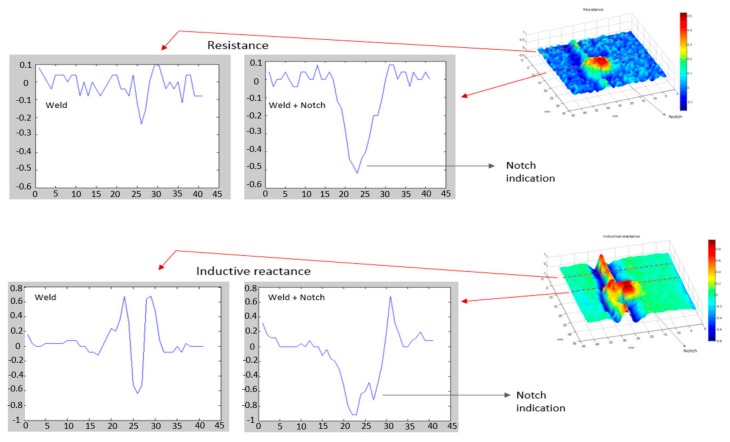
The notch presence amplifies the resistance signal, while in the inductive reactance, the notch can be easily identified through the appearance of a second peak attached to the weld signal.

The testing sample was scanned with different scan speed using the orthogonal EC transducer operating at 400 kHz. The first experiment was performed to evaluate the transducer capability detecting the notch at a low speed condition of 0.05 m/s. Figure 9 and Figure 10 present the inspection results in terms of the resistance, and inductive reactance components of the complex impedance obtained by the automated scan. One can observe that the notch indication was clearly separated from the weld bead on both impedance components, presenting a signal-to-noise ratio of 14 dB. This result confirms the potential of the EC with coils’ orthogonal configuration for detecting cracks in welded joints, as reported earlier [11,12,16,18].

In order to clearly understand the signal signature, two EC signals from the scanning result were selected for analysis. One signal from the weld with no influence of the notch was chosen, along with one signal with the influence of the weld and the notch. As can be noticed, the notch presence amplifies the resistance signal, while in the inductive reactance, the notch can be easily identified through the appearance of a second peak attached to the weld signal.

**Figure 11:**
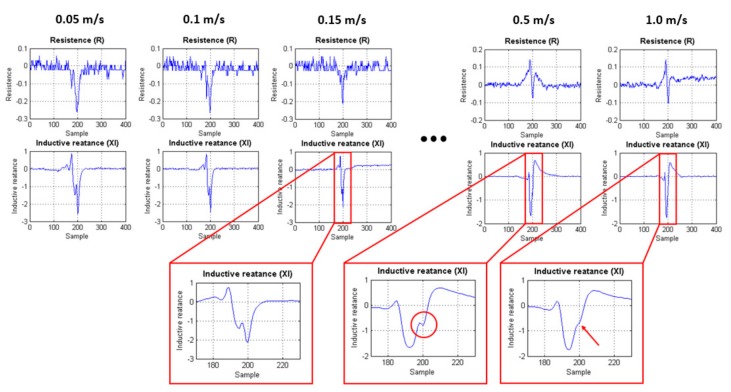
Different speed tests inspection. In red is highlighted the notch indication in velocities of 0.5 and 1.0 m/s.

The inspection speed is an important parameter for the present study, because once the in-line inspection tool carrying the sensors navigates inside the pipe, it is propelled by the fluid under production (petroleum, gas, etc.), and the fluid flow determines the tool speed in field situation. Therefore, a fast inspection tool results in a shorter inspection process, which means higher production during inspection and a shorter interference in the production original course. Considering such issues, Figure 11 presents different inspection speed tests, varying from 0.05 to 1.0 m/s, of a single transducer passing over the weld with the notch. In the results, the notch signal is aligned at sample 200. As mentioned, the notch presences amplify the signal of the resistive component, while in case of the inductive reactance, a second peak in the weld signal is verified, being very prominent in the low speed tests of 0.05, 0.1 and 0.15 m/s. However, when the inspection speed is increased, such variation is not very evident.

For high-speed tests, 0.5 and 1.0 m/s, it is possible to notice a different behavior in the resistance and reactance signal. The resistance signal attenuates mainly because of the intense physical impact of the transducer in the weld root penetration, which is negligible in the lower speed tests. Besides that, the notch peak in the inductive reactance signal starts to merge in the weld signal, due to the fact there is less inspection data. It is important mention that the hardware data rate was the same for all speed conditions, 100 Hz (achieved with Goertzel algorithm), but as the speed test is increased, the longitudinal scan resolution decreases. At 0.5 m/s, the red circle indicates the attenuated notch peak adjacent to the weld signal, while at 1.0 m/s, the red arrow indicates a smooth deformation in the weld signal caused by the notch presence. Thus, the higher the speed test, the stronger the transducer impact in the weld penetration, which disturbs and attenuates the notch signal, and the lower the inspection longitudinal resolution.

The hardware data rate is a relevant parameter for high-speed tests, and the implementation of Goertzel algorithm enhanced the processing time of the impedance evaluation, however, the data streaming still need to be improved to achieve a better identification of the notch at 1.0 m/s.

**Figure 12:**
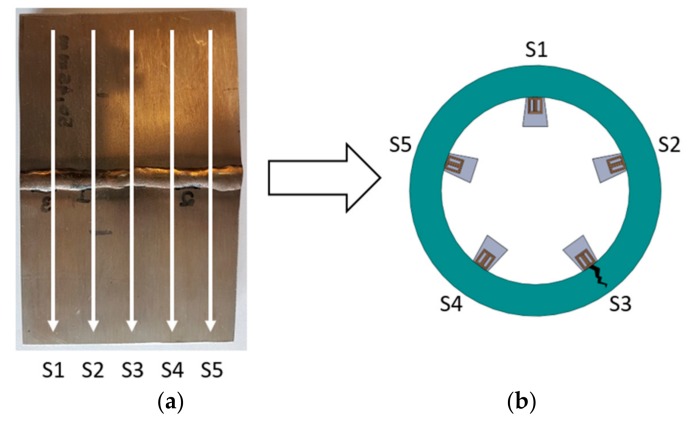
Five orthogonal eddy current (EC) transducer represented by S1, S2, S3, S4 and S5, scanned the clad sample with the EDM notch. (**a**) The arrows indicate the scanning direction; and (**b**) representation of the circumferential condition to inspect clad pipelines.

Finally, to verify the feasibility of implementing an internal inspection tool to detect fatigue cracks in clad pipelines, was tested an array of five EC transducers scanning the clad sample at 0.5 m/s. The sensors were excited, and their response signal measured using the hardware developed. To scan the sample, the sensors were set in longitudinal alignment with a lateral spacing of 7 mm (Figure 12a), reaching the high scan resolution for in-line inspection tools defined by Barbian et al. [20]. Although the tests were performed on a flat plate, it is considered representative for a circumferential inspection condition, as illustrated in Figure 12b. The flat arrangement is used mainly because it simplifies the mechanical support of the sensors and the tests with different scan speed.

The challenge associated with the notch detection at high-speed condition can be minimized once the weld signal signature is considered, similar for a given speed condition. This consideration brings the possibility to subtract the weld signal from the inspection data, in order to highlight the notch presence. Therefore, before the EC array inspection, several measurements of the weld signal were made in order to have a reference weld signal. Such reference signal was subtracted from each EC transducer’s signal (S1, S2, S3, S4 and S5).

**Figure 13:**
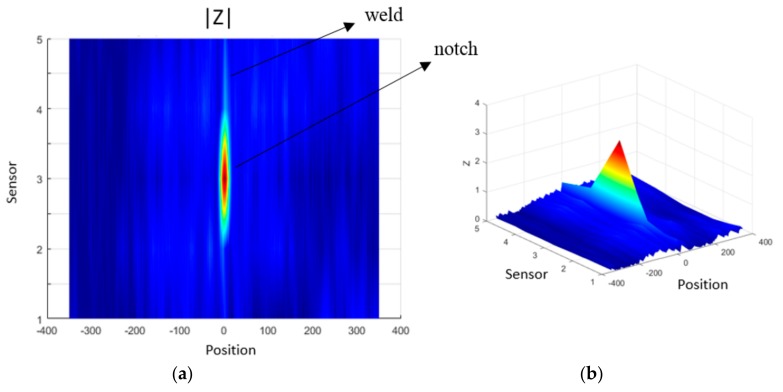
Scan maps of the inspection results in terms of the impedance magnitude of the array test in a relevant speed condition, 0.5 m/s. (**a**) 2D plot of the inspection result with the notch and weld bead indication; and (**b**) 3D visualization of the inspection result.

Figure 13a,b show scan maps of the transducer inspection results with the weld signal subtracted. The scan maps were obtained from the interpolation of the inspection signals of each transducer, where it is possible to identify the notch presence by the result of the impedance magnitude in a speed relevant for field application, 0.5 m/s. The five transducers are indicated in the “*y*” axes, and they scanned along the clad sample, where the position “zero” was set in the center of the sample. The presented result shows a significant repeatability during the tests, and confirms the potential of the EC system to inspect clad pipelines.

## 4. Conclusions

The weld root of a clad sample was inspected with an orthogonal EC transducer and the EDM notch was clearly identified, corroborating the excellent results reported in the referred bibliography.

A dedicated hardware was developed to drive the EC transducer and measure the electrical complex impedance. The Goertzel algorithm implementation improved the hardware data rate, which seems to be a good alternative for high-speed inspection tools where only a single frequency is evaluated.

Different inspection conditions were tested and as the speed is increased, the notch identification starts to merge in the weld signal, requiring, though, a higher data streaming from the electronic hardware to perform inspections above 1.0 m/s.

The suggested EC system using the developed hardware and the transducer with orthogonal configuration presented the possibility to implement an in-line inspection tool to detect fatigue cracks in clad pipelines.

## References

[1] Jones R., Karunakara N.D., Mair J., Wang H. Reeled Clad SCR Weld Fatigue Qualification. Proceedings of the Offshore Technology Conference.

[2] Largura J. (2011). Prediction of Fatigue Behavior of Seal Welds of Lined Pipes. Master’s Thesis.

[3] Da Silva S.E. (2010). Fatigue Crack Propagation in Circumferential Welded Joints of Steels for Rigid Risers Class API 5l Grade X80.

[4] Freire J. (2009). Engenharia de Dutos.

[5] Doosan Babcock Energy Limited (2009). Evaluation of the Effectiveness of Non-Destructive Testing Screening Methods for In-Service Inspection.

[6] Albright A. (2007). The Detection of Stress Corrosion Cracking in Natural Gas Pipelines Using Electromagnetic Acoustic Transducers. Master’s Theses.

[7] Ginten M., Penny T., Richardson I., Russel Q. (2014). Integrity Management of Stress Corrosion Cracking in Pipelines—An Integrated Approach.

[8] Reber K., Beller M. (2003). Ultrasonic In-Line Inspection Tools to Inspect Older Pipelines for Cracks in Girth and Long-Seam Welds.

[9] Cheng W., Shiwa H., Yoneyama H., Huang H., Takagi T., Uchimoto T. Ultrasonic and eddy current testing of defects in Inconel welding metals. Proceedings of the 12th MAGDA Conference.

[10] Bouda B., Lebaili S., Benchaala A. (2003). Grain size influence on ultrasonic velocities and attenuation. NDT E Int..

[11] Yusa N., Machida E., Janousek L., Rebican M., Chen Z., Miya K. (2005). Application of eddy current inversion technique to the sizing of defects in Inconel welds with rough surfaces. Nucl. Eng. Des..

[12] Yusa N., Janousek L., Chen Z., Miya K. (2005). Diagnostics of stress corrosion and fatigue cracks using benchmark signals. Mater. Lett..

[13] Todorov E., Nagy B., Levesque S., Ames N., Na J. Inspection of laser welds with array eddy current. Proceedings of the AIP Conference.

[14] García-Martín J., Gómez-Gil J., Vázquez-Sánchez E. (2011). Non-Destructive Techniques Based on Eddy Current Testing. Sensors.

[15] Huang H., Yusa N., Miya K. Eddy current testing and sizing of fatigue cracks. Proceedings of the 12th A-PCNDT 2006—Asia-Pacific Conference on NDT.

[16] Lamtenzan D., Washer G., Lovez M. (2000). Detection and Sizing of Cracks in Structural Steel Using the Eddy Current Method.

[17] Xie R., Chen D., Pan M., Tian W., Wu X., Zhou W., Tang Y. (2015). Fatigue Crack Length Sizing Using a Novel Flexible Eddy Current Sensor Array. Sensors.

[18] Watkins D., Kunerth D.C. Eddy Current Examination of Spent Nuclear Fuel Canister Closure Welds. Proceedings of the 2006 International High Level Radioactive Waste Management Conference (IHLRWM).

[19] Tan L. (2008). Digital Signal Processing: Fundamentals and Applications.

[20] Barbian A., Beller M., Hartmann S., Schneider U. Resolution Ultrasonic In-Line Inspection: Added Value and Special Applications. Proceedings of the 6th Pipeline Technology Conference.

